# Dentistry vs. Money

**Published:** 1871-09

**Authors:** R. S. Wells


### DENTISTRY *vs.* MONEY.

BY R. S. WELLS.

Read before the Wisconsin State Dental Society, July 12, 1871.

That "self protection is the first law of our being," is the universal voice of reason, and "if any provide not for his own, and especially for those of his own house, he hath denied the faith, and is worse than an infidel," is the word of inspiration, each agreeing to the same truth.

To provide for our professional wants, and to protect our mutual interests, is the object of our gathering in associate capacity to-day.

I have chosen my theme, "Dentistry *vs.* Money," or "Values in Dentistry," for the following reason:

Subjects pertaining to the *acquirement* of professional knowledge and skill, are the themes almost exclusively dwelt upon by our writers and public teachers.

I would not underrate these themes, for it is of the first importance that we *have* skill and knowledge, but it is essential also, that we know how to *use* them. A defective bolt may destroy the efficiency of the most perfect engine, and so may a misconception of business principles applied to skill and knowledge, render them unproductive of pecuniary reward.

In adopting this, our chosen calling, without doubt, the monitary idea was the principle one with us all, then why pass it by in our public deliberations, with such silent indifference, while in our private meditations, it claims so large a share of our thoughts.

Yankees worship success, and especially devout are they, at the golden shrine of pecuniary success. "The surest way to be a success, is to succeed," say they. Morals in *methods* are ignored. Let us not fall in with this pernicious doctrine, but use only right and honorable means of advancing our prosperity.

When I see a pecuniarily successful dentist, I want to "button-hole" and learn from him the secret of it.

We are in the habit of hearing this statement in answer to our inquiries on this point: "The skillful will *always* succeed. But the question arises, How about opportunity? If they mean by skill, ability to take advantage of circumstances, or to *make* opportunity, I grant it, for it is the secret of all success.

> "Fortune is a lazy goddess,
> She will never come to you."

But if they mean mere professional knowledge and skill, I deny it, for it is well known that the amount of money a man may have, is not always in the ratio of his ability. Circumstances may favor one and not another. Look, for illustration, at Goodyear and Bacon. The former had inventive talents of the highest order, which is producing a rich pecuniary harvest; but he sowed, while Josiah reaps, and we are his golden sheaves.

A merchant with sufficient capital and ability, to buy advantageously, and sell cheaply, and with a good market—the main conditions of success—may utterly fail, because he neglects other conditions, miner in themselves, in not securing a good location for trade, or in not advertising properly. I do not counsel greater greed for gain, but better methods for obtaining it.

There are certain conditions to be met in all kinds of business, and a proper understanding of those pertaining to our specialty, is of interest to us all.

All legitimate and honorable business is founded on the principle of reciprocity, or value returned for value received. A man must have an article of value to exchange for other values, and then must find or *make* a market for it to succeed. I say make a market, for to create a demand for his article, may require business skill of the highest order; but this will be considered more fully under the head of advertising.

What is value? "Value consists in use."

Says an eminent writer on National Economy, "It is that property or those properties which render anything useful. They are valuable, because they are useful. A reaper is valuable for its useful qualities. The same is true of ornaments, they are valuable because they are useful for ornamental purposes." The same is true of knowledge, it is valuable because it is useful to alleviate suffering, shield us from danger, and add to our happiness.

So of everything necessary to the support and comfort of man. They are valuable because of their useful qualities.

Now, what have we of value to exchange for money? The first thing I will mention is

SKILL.

When your skill puts into a persons mouth, a thing of beauty, more valuable for ornamentation than the costliest diamond, that rejuvinates the fading charms of the waning beauty more than the most costly toilet, that removes facial deformity as no other art however costly, or surgical device however expensive can, that gives more gustatory pleasure than the most luxurious viands, that adds to the length of the days of the invalid, by aiding nature in her work of digestion, as nothing else does, then for *such* a product of skill, to charge no more than a blacksmith receives for the same amount of time expended on your horse, looks, certainly, as though you thought your product had no more value than his. Suppose, if you find a diamond by an hour's search, is that a reason why you should sell it for the price of an hour's wages?

A man is suffering the pangs of toothache, by your skill you relieve him in an hour, or it may be in a minute, shall you therefore be compensated only for an hour's or a minute's time at day laborers' wages? Clearly not. The less the time, the more the pay. There is sometimes, not always, a realization of this idea in surgical dentistry, but when we look at mechanical dentistry, as it is commonly called, how false are the views of many; they charge as though they were mere mechanics, and the result is, the public take them at their own estimates of themselves, and their own operations show sooner or later, the bad effect of this low standard. A man is never up to his ideal, but always below it, therefore correct ideas are all important on this, as well as on other subjects, to make our practice right.

### KNOWLEDGE.

The next thing we will consider, is the value of dental knowledge. Any respectable attorney will charge a fee of from ten to fifty dollars for an ordinary case. This retainer is a prepayment for the legal knowledge he expects to impart.

If he pleads in any court for you, this service will call for an additional fee. In fact, his fees are mainly for legal knowledge imparted, and so it is with the medical profession. They no longer furnish medicines of value, but charge for medical knowledge mainly. But how about the dentist. Shall he furnish advice that will save priceless gems for future beauty and usefulness, and receive no compensation for that knowledge? It has cost him much in time and money for its acquirement, not only the first investment for tuition, but for the experience of after years, which comes through study and experiments. He must spend no small amount of time in the investigation of new facts and theories to keep pace with the rapid strides of our vigorous young profession.

A wealthy patron of an artist, once complained to him of charging what appeared to be a large sum for ten days work. The artist replied, "It took me ten years to learn to paint that picture in ten days."

### SYMPATHY AND PATIENCE.

Shall we receive a compensation in money for these, asks some of you. Why not? Who of you do not know by experience how completely his whole body may be drained of its nervous forces, by a demand at the chair for a few hours for sympathy and patience, by a young or refractory patient, yet how valuable is it to them, and to all in hours of distress. A visit to a dentist for the first time, is a great event to many. The most who come to us are in pain, or at least are suffering from a nervous dread of our operations; kind words and gentle manners are appreciated then as at no other time. If you are harsh and unfeeling or impatient, you repel them from you, but if you show a delicacy of feeling for their fears, and have soothing words for their sufferings, and are patient with them in their fretful and often unreasonable moods, they will treasure up a kind remembrance of you, which will go far toward removing the disagreeable sensations they experience at your hands, and which you may turn into gold. More than once have I known patients leaving a skilled but harsh operator, to employ one of less skill, but of known gentleness and kindness.

### CHEERFULNESS.

This, too, has its value; for many a heavy heart is carried into your presence, which a cheerful grateful word will help to lighten. You need not be frivolous or boisterous, but a well stored mind can gently lead the troubled one to other objects than self for contemplation, and thus may you rob your office of half its terrors.

Let nothing in the display of instruments, anatomical preparations, or in your conversation suggest somber thoughts, but rather let pictures, books, and flowers direct the mind with your help, to something to think of, rather than its present fears.

This quality, then, has especial valuc to you, because of its peculiar usefulness.

### A GOOD ADDRESS.

By this is meant the ability to say the right thing at the right time and place. It is an outgrowth of refinement; however, a person may be truly refined in feeling, and yet lack the ability to express his thoughts skillfully. This is a thing for cultivation. You have golden opportunities while a patient is under your care for treatment, to impress them with the importance of dental surgery and hygiene.

That there is a vast amount of ignorance in the teachings of our science, we well know from daily experience; is it not, therefore, a duty we owe the public, and especially our patrons, to enlighten them? Will we not be public benefactors, if we induce them to take the ounce of prevention rather than the pound of cure?

With the class who *intend* to take proper care of their dental organs, their are probably more teeth annually lost from neglect, than are saved by timely operations. If this is true of those who are comparatively enlightened, what shall we say of the masses who never think or care for these things. Could we secure this last named class for our patrons, our business would vastly increase, besides we would confer lasting benefits on them.

### NEATNESS.

What! Coin this into money?

If it is useful in suppling a want, it is valuable.

This is a day of judging by appearances; therefore, appearances can not be neglected.

To use a common phrase, there is money in it. Be scrupulously neat, not only for appearance sake, but for the *virtue* there is in it. Let no bad odors escape from your breath, your hands or your office, else a suspicion is excited against you in the minds of the refined, that will tell against your pockets.

A common maxim is, "Dress bespeaks the man." The discriminating judge, and that rightly, that he who is slovenly in his habits will be slovenly in his operations.

A dentist whose office is furnished neatly or even elegantly, can get larger fees than he can for the same services rendered in dirty, shabby rooms.

Let books, pictures, flowers and works of art, adorn your office, and it will attract the refined classes to you. The shoddy van are attracted by mere appearances, while the crowd follow when these two classes lead.

### ARTISTIC TASTE.

By artistic taste is meant that which pertains to the skillful use of form, proportion, beauty, curvature and color.

It is the outgrowth of culture.

The mechanic works by fixed rules, does as he is taught, and the product of his hands is mechanical. In other days, a branch of our business was called, and that rightly, *mechanical* dentistry. Some have not outgrown the idea yet, and are still mechanics, content to work for mechanic's wages. They have no idea of anything going into the product of their hands, higher than mere labor. Agents who have visited every office in the country, report that this class, in some sections, have been obliged to clip expenses, here and there, to make their ends meet, till they are far poorer than ordinary mechanics about them. They have talked "cheap dentistry," till *they have created a demand* for it, and they have also in some instances secured a reputation. Such a reputation, of being CHEAP DENTISTS, with its necessary concomitant poverty, to bless them for their wisdom all their days.

But there is another class who understand wherein their *true* interest is, and have taken a different course. They have cultivated the artistic spirit by long years of study, till they are enabled to breathe that spirit into all they do. Dead blocks of porcelain, by their touch are things of life and beauty, rivaling nature in graceful curves, delicate colors, beautiful forms, and nice proportion. The refined and cultivated appreciate this, and are willing to pay for it. This class make or unmake our reputations. They with all sensible persons, are suspicious of a professedly good article, that is cheap, for fear it has no value.

It is optional with each whether he will bring an artistic product to a good appreciative market, or a cheap mechanical product to a poor market.

### ADVERTISING.

This subject does not properly belong to my theme, but being collateral to it, and intimately connected with our own interest, I choose to say a few words on its use.

What collossal fortunes are made by the skillful use of the press. In fact, it is the main element of success with the quack. He blows his brazen trumpet loud and long till the credulous flock to be fleeced. It seems to me that *this is an element worth redeeming from the vicious uses of empiricism.*

If the press enables worthless things to bring such rich pecuniary harvests, should there be any hesitancy in using it to proclaim the merits or make a market for a good thing? There needs to be wisdom used in this as in other things. Mere puffing is of doubtful expediency, but the most fastidious on this subject can not object to the enlightening of the masses on subjects pertaining to our specialty, and to teach them wherein is their highest interest, for their interests are identical with ours in this matter.

Let me suggest that this society offer a prize for the best written tract, or tracts, or books, on subjects pertaining to the care and preservation of the teeth; let the matter be put into the hands of a committee to report at the next meeting of the society, and if they are adapted to the public want, let them be printed under the auspices of this society. Can it engage in a better work? Will it not do more to regulate the practice of dentistry than the best devised laws? Give the public the means of discriminating between the good and bad, and their interests will lead them to the man that will give them the most value for their money.

I will close, by giving you in his own words,

"PAT'S VIEWS OF TOOTH PULLING."

Och, doctor, me darling, just come here a minnit,
  I've got a bad tooth, and the divil is in it;
It keeps such a hoppin and jumpin about,
  I think on my soul, I must have the thing out.

Och! now *do* be careful; 'tis uncommonly tinder,
  Oh! Mother of Moses!! Oh!!! Murther!!!!
Is it out? Faith, I thought you for sartin,
  The top of my head from my body was partin.

The sinsation, I say, was uncommonly quere,
  As though a big engine went in at each ear,
And met each the other, with a tirrible crash,
  Which sent all their fixins to gineral smash.

And now for this torture, I suppose I must pay,
  How much is it sir? A half dollar, did you say
It's too much for a half minutes work,
  You ought to pay me, 'twas earned with a *jirk*
The blacksmith will pull thirty minutes, and is illing
  To pull thirty more, and he'll charge but a shillin.

